# *In vitro* Characterization of the Rapid Cytotoxicity of Anticancer Peptide HPRP-A2 through Membrane Destruction and Intracellular Mechanism against Gastric Cancer Cell Lines

**DOI:** 10.1371/journal.pone.0139578

**Published:** 2015-09-30

**Authors:** Jing Zhao, Xueyu Hao, Dong Liu, Yibing Huang, Yuxin Chen

**Affiliations:** 1 Key Laboratory for Molecular Enzymology and Engineering of the Ministry of Education, Jilin University, Changchun, China; 2 School of Life Sciences, Jilin University, Changchun, China; 3 National Engineering Laboratory for AIDS Vaccine, Jilin University, Changchun, China; Wayne State University School of Medicine, UNITED STATES

## Abstract

In this study, HPRP-A2, a synthetic 15-mer cationic peptides with all D-amino acids, effectively inhibited the survival of gastric cell lines in a dose-dependent manner. Gastric tumor cells killing by HPRP-A2 involves a rapid collapse of the membrane integrity and intracellular pathways. Propidium iodide (PI) and lactate dehydrogenase (LDH) assays demonstrated that one-hour treatment with HPRP-A2 led to membrane permeability changes of BGC-823 cells in a dose-dependent manner. Moreover, HPRP-A2 induced apoptosis in BGC-823 cells involves a marked increase in generation of reactive oxygen species (ROS),caspase-3, -8 and -9 activation, a reduction of mitochondrial membrane potential (MMP), and cell cycle arrest in G1 phase. In addition to its inherent cytotoxicity, HPRP-A2 synergized strongly with doxorubicin (DOX) to enhance the efficacy of killing gastric tumor cells i*n vitro*. We believe that HPRP-A2 with all D-amino acids could be a potent candidate of anticancer therapeutics, especially in combination therapy.

## Introduction

Over past decades, although breakthroughs have been achieved in the development of cancer therapies, resistance and nonspecific toxicity of conventional drugs are still bottle-neck issues for potential clinical practices [1–3]. Hence, it is urgently required to develop novel drugs with different modes of action which can overcome the shortcomings of many available drugs.

Currently, the potential applications of anticancer peptides (ACPs) as therapeutic agents for the treatment of cancer progression attract more attention than conventional chemotherapy mainly because of the following properties: (1) high specificity. The positively charged peptides selectively target cancer cells that carry negative charges and have different membrane components from normal cells [4, 5];(2) novel mode of action. It could avoid established multidrug-resistance mechanisms [5–7]; (3) synergistic anticancer effect with chemotherapeutics. It has been reported that certain ACPs can produce synergistic anticancer activity when combined use with different conventional anticancer drugs [8–10].

The general mechanism of peptide-induced cell death is cytoplasmic membrane disruption via micellization or pore formation, although some of ACPs are reported to trigger apoptosis by death receptor pathway and/or mitochondrial pathway [8, 11]. Moreover, the pore formation on the cell membrane and the change of the membrane permeability may provide a better channel for the entry of other anticancer drugs into cells and enhance their anticancer activities [8, 12].

Our previous study has proven that HPRP-A2 can induce the cell death and simultaneously enhanced DOX/epirubicin (EPI)-induced apoptosis in HeLa and HepG2 cell lines [12]. In addition, due to the D-amino acid composition, HPRP-A2 is resistant to proteolytic cleavage and retains equivalent anticancer activities to its L enantiomers [6, 12]. Based on the previous studies, we aim to accomplish two objectives in this study: to delineate the underlying anticancer mechanism of HPRP-A2 and to investigate the synergistic anticancer effect on BGC-823 and SGC-7901 cells when combined HPRP-A2 with DOX.

## Materials and Methods

### Cell lines and cell culture

Human gastric cancer cell lines BGC-823 and SGC-7901 were obtained from the American Type Culture Collection which authenticates the cell lines by short-tandem repeat DNA testing, were used within 6 months of resuscitation and grown in DMEM with fetal bovine serum (FBS; 10% v/v), penicillin (100 U/ml), and streptomycin (100 U/ml) in a humid atmosphere at 37°C with 5% CO_2_.

### Peptide synthesis and purification

The peptide was synthesized by the solid-phase peptide synthesis using Fmoc (9-fluorenyl-methoxycar-bonyl) chemistry as described previously [13]. Further characterization was detected by mass spectrometry and amino acid analysis. DOX·HCl was purchased from Meilun Biology Technology Co., Ltd. (Dalian, China).

### Cell viability assays

BGC-823 and SGC-7901 cells (5×10^3^) were plated in triplicates in 96-well microtiter plates. Complete medium was replaced after 24 h with 100 μl of fresh medium containing various concentrations of drugs. After a further 24 h, cells were incubated with 3-(4,5-dimethyl-2-thiazolyl)-2,5-diphenyl-2H-tetrazolium bromide(MTT)at 37°C for 4 h. Thereafter dimethyl sulfoxide (DMSO) was added to dissolve the formazan crystals and the absorbance at 492 nm was measured with a microplate reader (GF-M3000; Gaomi Caihong Analytical Instruments Co., Shandong, China). Jin’s formula was used to further quantify the synergistic effect of the combination treatment of HPRP-A2 and DOX. The formula is: Q = Ea+b / (Ea + Eb—Ea × Eb), where Q is the combination index; Ea+b represents the cell proliferative inhibition rate of the combined drug; Ea and Eb are signs of the cell proliferative inhibition rate of each drug. After calculation: Q<0.85, Q>1.15 and 0.85<Q<1.15 indicate antagonism, synergy, and additive effect, respectively [14].

### Hemolysis activity assay

Hemolysis activity analysis was performed as described previously [13]. To obtain red blood cells, fresh human blood stabilized with EDTAK was centrifuged at 1,000 rpm for 5 min, washed twice with PBS and diluted to a final concentration of 2% in PBS. 70 μL of 2% human erythrocytes were added to a round-bottomed 96-well plate, followed by 70 μL of different concentrations of HPRP-A2. After incubation at 37°C for 1 h, the plate was then centrifuged at 3,000 rpm for 10 min and 90 μL of supernatant was transferred to a flat-bottomed 96-well plate. The release of hemoglobin was determined by measuring the absorbance of the supernatant at 540 nm. Erythrocytes in PBS and distilled water (dH_2_O) were used as negative and positive controls, respectively. The hemolytic activity was calculated as the percentage of experimental group and positive control, after subtraction of negative control respectively. Data are the mean ± SD of three independent experiments.

### PI assay

BGC-823 cells (1×106 cells/well) were seeded in six-well plates. After incubation with HPRP-A2 (5, 10, 15 μM) for 1 h, the cells were collected and then treated with 5 μg/ml PI at 4°C for 10 minutes in the dark. Cells were washed with PBS for three times and then detect the fluorescence intensity of PI using flow cytometry (FACSCalibur, Becton-Dickinson, San Jose, CA, USA).

### LDH release

LDH release activity was measured by LDH assay kit (Jiancheng Bioengineering, Ltd., China) according to the manufacturers’ instructions. BGC-823 cells were seeded at 5 ×10^3^ cells/well in a 96 well plate. After incubation with HPRP-A2 (5, 10, 15 μM) for 1 h, the release of LDH in the supernatant was measured with a microplate reader (GF-M3000; Gaomi Caihong Analytical Instruments Co., Ltd. Shandong, China) at 450 nm. Cells without treatment or lysed with triton X-100 was used as negative and positive controls, respectively. All experiments were carried out in triplicates. LDH activity was calculated as the percentage of experimental group and positive control, after subtraction of negative control respectively.

### ROS assay

ROS assay kit (BestBio, Co. Shanghai, China) was used to detect the generation of ROS. Cells (1×106) were treated by HPRP-A2 (5, 10, 15 μM) for 1 hour. The subsequent procedures were performed according to the manufacturer’s protocol. In brief, cells digested by trypsin were centrifuged at 1,500 rpm for 3 min, washed three times with PBS and then suspended in 500 μL PBS. After incubation with 2’,7’-dichlorofluorescin diacetate (DCFH-DA) fluorescent probe for 20 min at 37°C,cells were washed with PBS for three times and detected the green fluorescence intensity (in Geomean) by flow cytometry. The green fluorescence intensity was positively correlated with the level of ROS.

### Measurement of MMP

MMP was determined by the mitochondrial membrane potential assay kit with 5,5′,6,6′-tetrachloro-1,1′,3,3′-tetraethylbenzimidazolcarbocyanine iodide (JC-1), which is a probe of mitochondrial activity (BestBio, Co., Shanghai, China). JC-1 is always used to detect mitochondrial depolarization occurring in the early stages of apoptosis. When cells with high MMP, JC-1 gathered in the mitochondrial matrix, forming J-aggregates and produce red fluorescence. Conversely, cells containing JC-1 monomer monomer monomermonomer have low MMP and show green fluorescence. Mitochondrial depolarization was determined by a decrease in the red (590 nm, FL-2 channel)/green (530 nm, FL-1 channel) fluorescence intensity ratio. Briefly, after treatment with HPRP-A2 (5, 10, 15 μM) for 1 h, BGC-823 cells were collected and incubated with 0.5 ml JC-1 working solution for 20 min at 37°C, then washed twice with PBS, suspended in PBS, and analyzed by flow cytometry (FACSCalibur, Becton-Dickinson, San Jose, CA, USA).

### Caspase activity assay

Cells were treated with HPRP-A2 (10, 15 μM) for 24 h and then levels of caspase activities were measured using the corresponding caspase activity detection kits (BestBio, Co., Shanghai, China), according to the manufacturers’ instructions. Average data are presented as the mean ± SD of at least three independent experiments.

### Cell cycle analysis

BGC-823 cells (1×10^6^) were seeded in 6-well plates for 24 h. After induction with HPRP-A2 (5, 10, 15 μM) for 24 h, cell cycle distribution was determined by a FACScan cytometer and Cell Quest software (FACS Calibur, Becton-Dickinson, San Jose, CA, USA). All experiments were performed in triplicates.

### Statistical analysis

The data are presented as means ± SD of three independent determinations. Statistical significance of differences between groups were analyzed by *t*-test, with significance accepted at *P* < 0.005 (*) and *P* < 0.001 (**).

## Results

### Peptide and cytotoxicity

**Fig 1:**
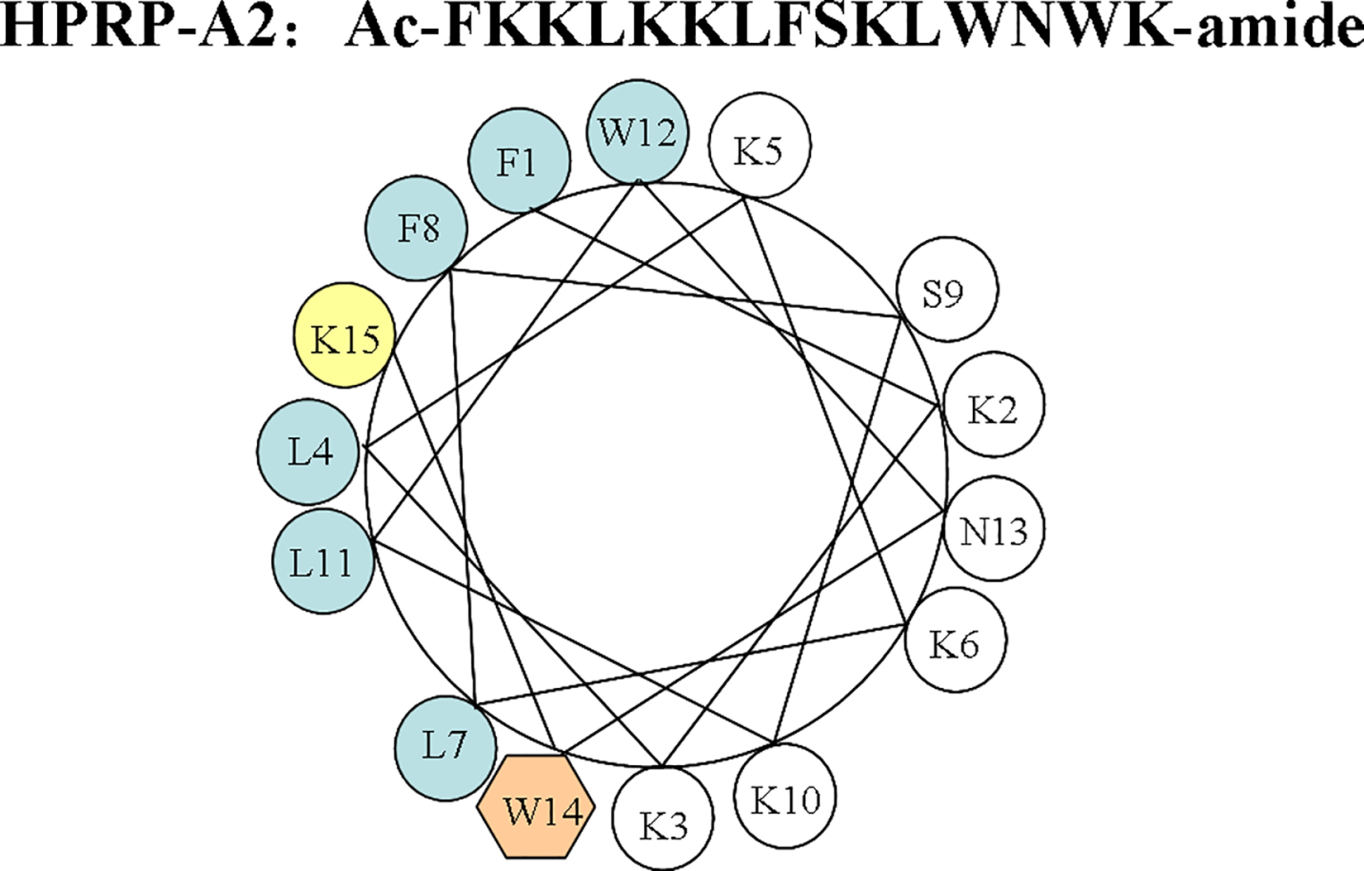
Peptide sequence and the helical wheel of HPRP-A2.

**Fig 2:**
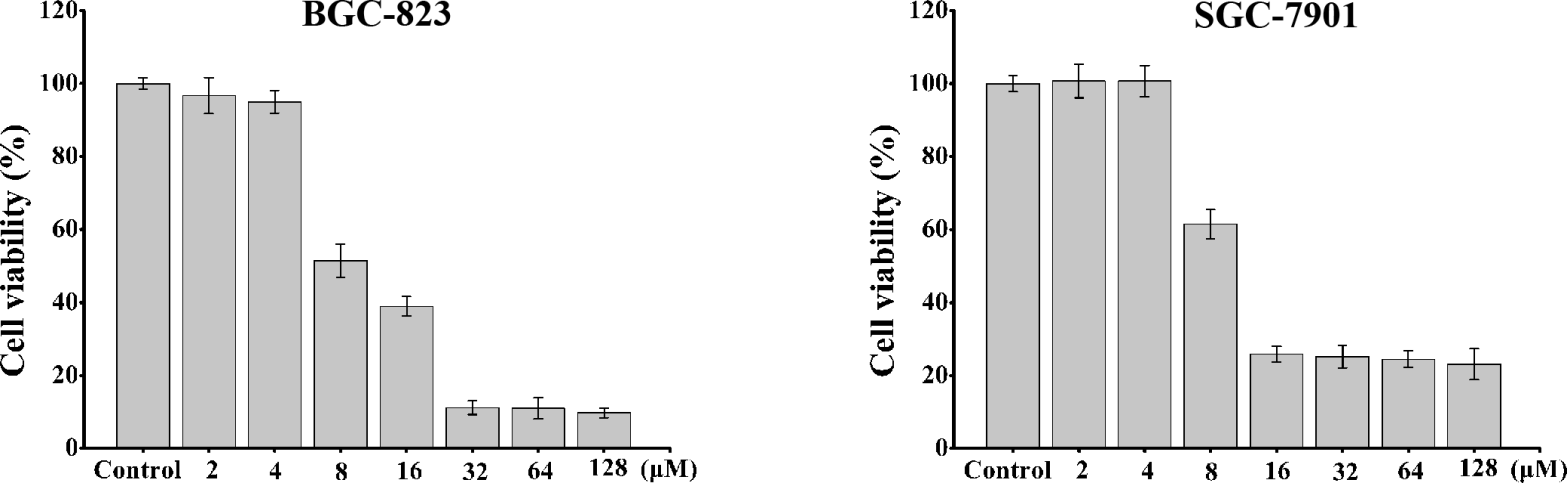
HPRP-A2-induced BGC-823 and SGC-7901 cell death. The cells were treated with different concentrations of HPRP-A2 for 1 hour. Cell viability was determined using the MTT method. Results are expressed as percentage of the control ± SD of three independent experiments. Statistical analysis compared the HPRP-A2 treatment group with the control groups (*P < 0.005; **P <0.001).

**Fig 3:**
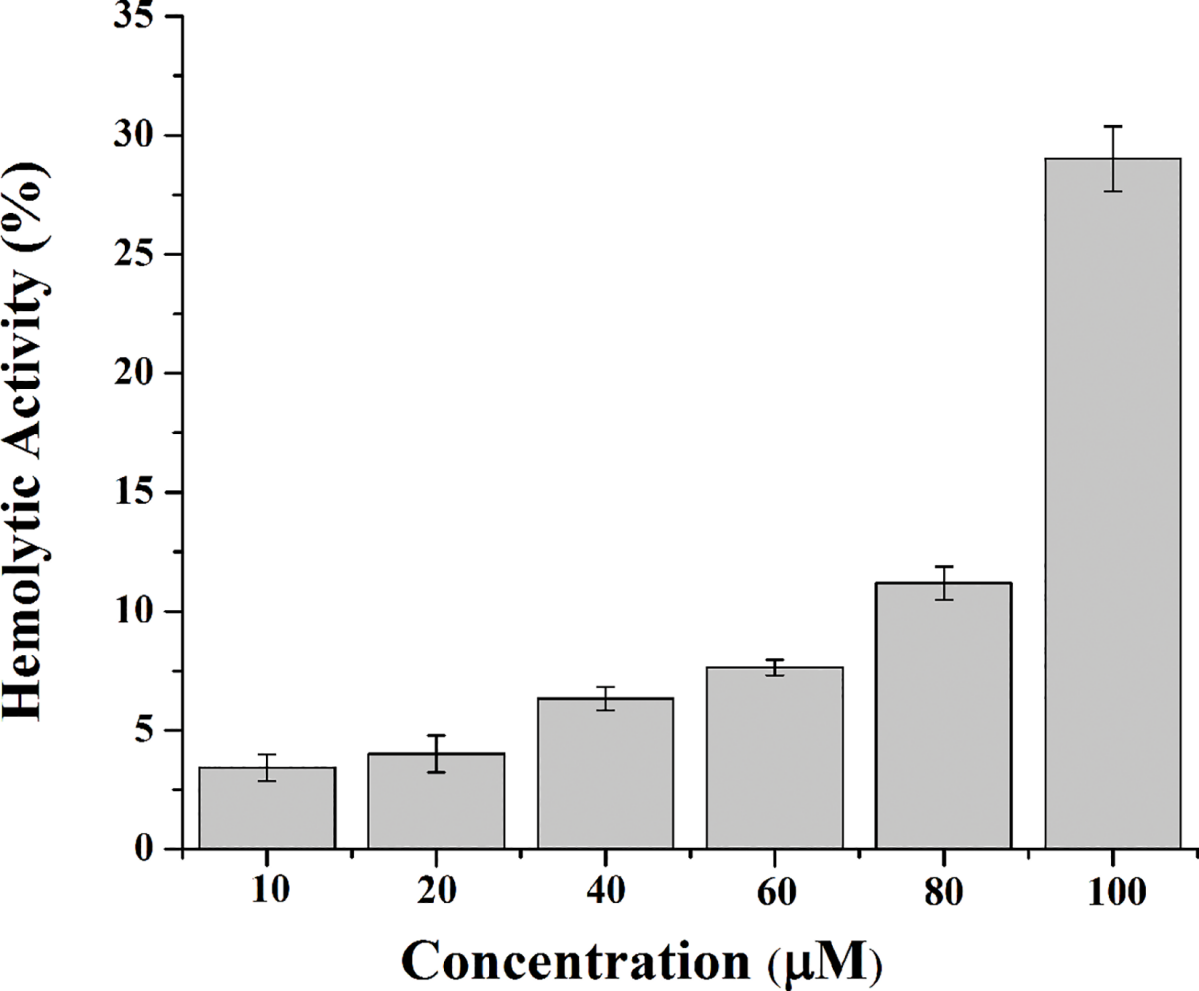
Hemolytic activity of HPRP-A2 against hRBCs. Data points present mean ± S.D. of three independent experiments. PBS and dH2O were used as negative and positive controls, respectively. Statistical analysis compared the HPRP-A2 treatment group with the positive control groups (*P < 0.005; **P <0.001).

As shown in Fig 1, peptide HPRP-A2 is a 15-residue α-helical amphipathic membrane-active peptide composed of all D-amino acids. Comparing the selective toxicity of HPRP-A2 towards gastric cancer cells and normal cells (human red blood cells), we can easily find that the IC_50_ (the concentration of drug at which cell viability was reduced by 50% compared with untreated cells) values are far less than the minimal hemolytic concentration (the concentration of drug that resulted in 20% cell hemolysis) of the HPRP-A2. These results indicated that HPRP-A2 can selectively kill the gastric cancer cells and spare the normal cells (Figs 2 and 3). Similar anticancer activities of the two cell lines (BGC-823 and SGC-7901) indicated that there was a broad-spectrum effect in the anticancer action of HPRP-A2. Owing to its membrane-active characteristic, HPRP-A2 shows the anticancer therapeutic potential since it is more selectively toxic towards tumor cells than normal cells.

### HPRP-A2 induced the enhancement of membrane permeability

**Fig 4:**
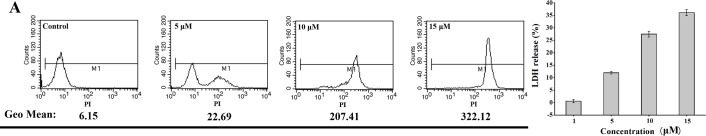
Membrane permeability changes of BGC-823 cells by monitoring PI and LDH. The cells were incubated with increasing peptide concentrations for 1 h at 37 oC. (A) Quantitative comparisons of fluorescence intensity (in Geomean) at various concentrations were analyzed by flow cytometry. (B) LDH in the supernatant was measured with a microplate reader at 450 nm. Cells without treatment or lysed with triton X-100 was used as negative and positive controls, respectively. LDH activity was calculated as the percentage of experimental group and positive control, after subtraction of negative control respectively (*P<0.005). Data are the mean ± SD of three independent experiments.

In order to verify the change of membrane permeability after incubation with HPRP-A2, the cellular uptake of PI and extracellular release of LDH were investigated with flow cytometry and microplate reader toward BGC-823 cells. As shown in Fig 4, the flow cytometric graphs of the PI move gradually to the direction of high fluorescence intensity in a concentration-dependent manner, and the increased release of LDH was also observed in the cells incubated with HPRP-A2. That is to say, HPRP-A2 could cause the damage of cell membrane and result in the enhancement of cell membrane permeability.

### HPRP-A2 caused the damages of mitochondrial function

**Fig 5:**
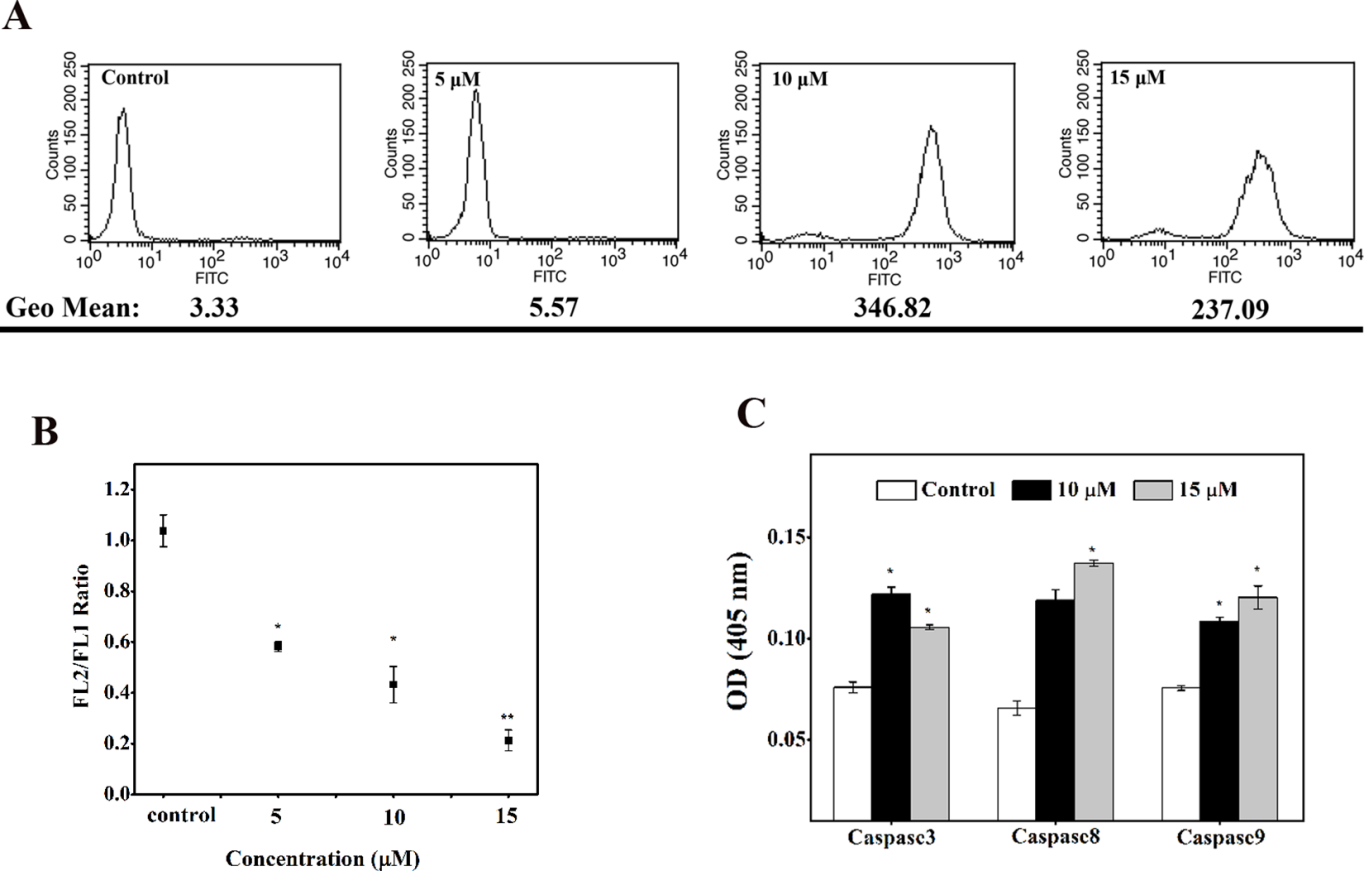
HPRP-A2-induced mitochondrial membrane potential. (A) BGC-823 cells were treated for 1 h and the generation of reactive oxygen species (ROS) was analyzed by FACS to quantitatively compare the fluorescence intensity (in Geomean) at various concentrations. (B) After 1 h treatment, the ratio of FL2-H/FL1-H decreased, indicating a reduction of MMP. (C) BGC-823 cells were treated with HPRP-A2 (10 and 15 μM) for 24 h and then levels of caspase activities were measured. Data are expressed as mean ± SD of three independent experiments. Statistical analysis compared the HPRP-A2 treatment group with the control groups (*P < 0.005; **P <0.001).

The intracellular reactive oxygen species (ROS) release and mitochondrial membrane potential (MMP) were detected with FACS to reflect the mitochondria function of BGC-823 cells *in vitro*. As shown in Fig 5A, the flow cytometric histogram of the cells incubated with higher concentration of HPRP-A2 revealed higher fluorescence intensity after incubation for 1 h. The corresponding flow cytometric quantitative comparison of fluorescence intensity in Geomean at these different concentrations showed a similar trend, namely that the increased release of ROS in BGC-823 cells in the presence of HPRP-A2 was concentration-dependent. Similar concentration-dependent changing trend was also observed in Fig 5B, the ratio of FL2-H/FL1-H markedly decreased, indicating a depolarization of MMP.

### Caspase activation and cell cycle analysis

**Fig 6:**
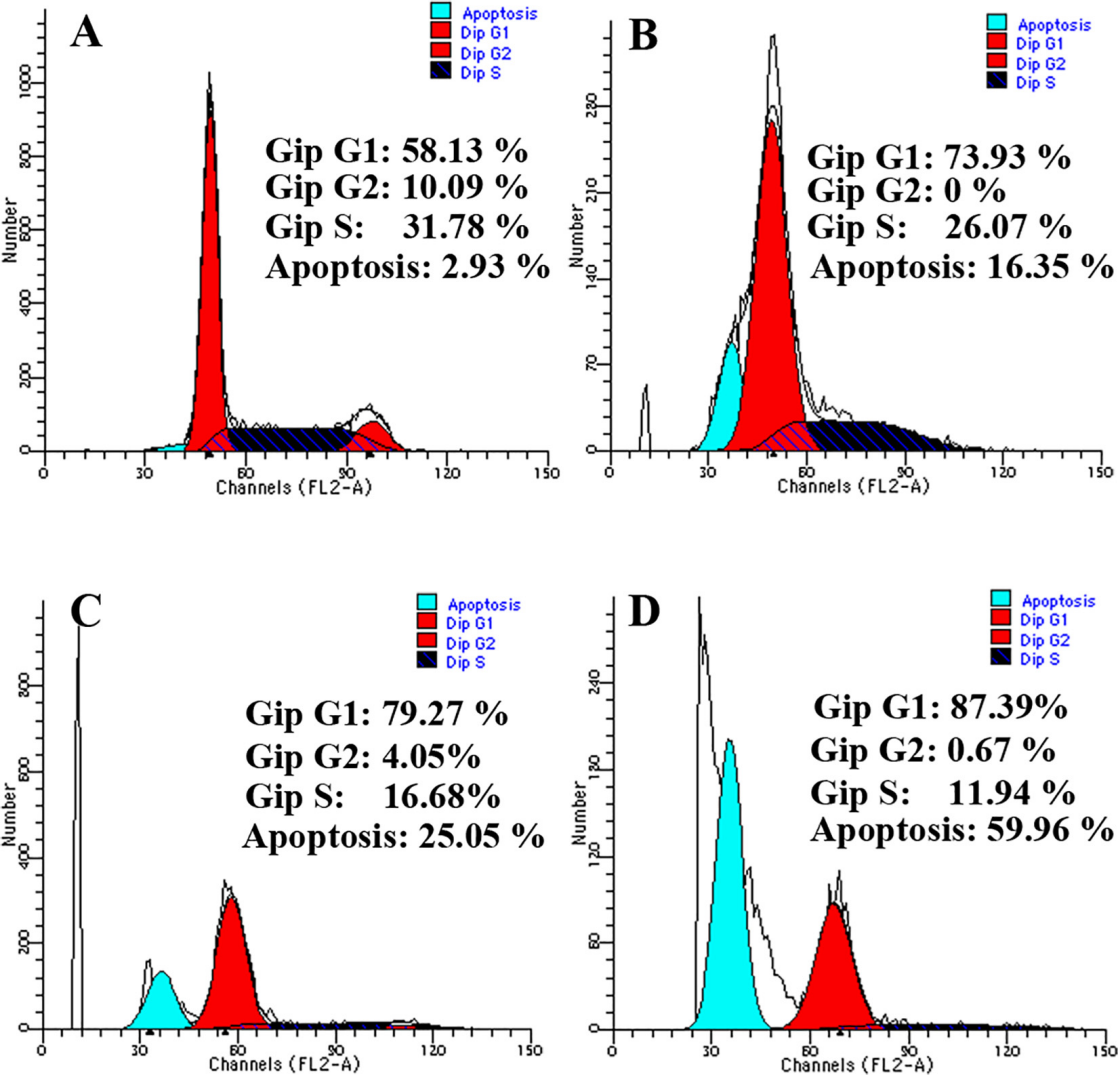
HPRP-A2 triggered cell cycle arrest. Cell-cycle distributions after treatment with 5, 10 and 15 μΜ HPRP-A2 for 24 h separately were detected by flow cytometry. Cell-cycle distributions were assessed by PI staining. The results were showed from one of three experiments with similar results.

Activation of caspase-3, -8 and -9 in HPRP-A2-induced cells was measured using a with a microplate reader at 405 nm. As shown in Fig 5C, caspase-3, -8 and -9 were all increased after treatment with HPRP-A2 for 24 h in BGC-823 cells,which suggests that the induction of apoptosis by the HPRP-A2 may be caspase-dependent. As shown in Fig 6, BGC-823 cells treated with the different concentrations of HPRP-A2 (5, 10, 15 μM) for 24 h resulted in the increases in sub-G1 arrest and in G1 arrest. These findings also strengthened the increased caspase-3 activity after the treatment of HPRP-A2.

### HPRP-A2-induced increase in DOX cytotoxicity

**Fig 7:**
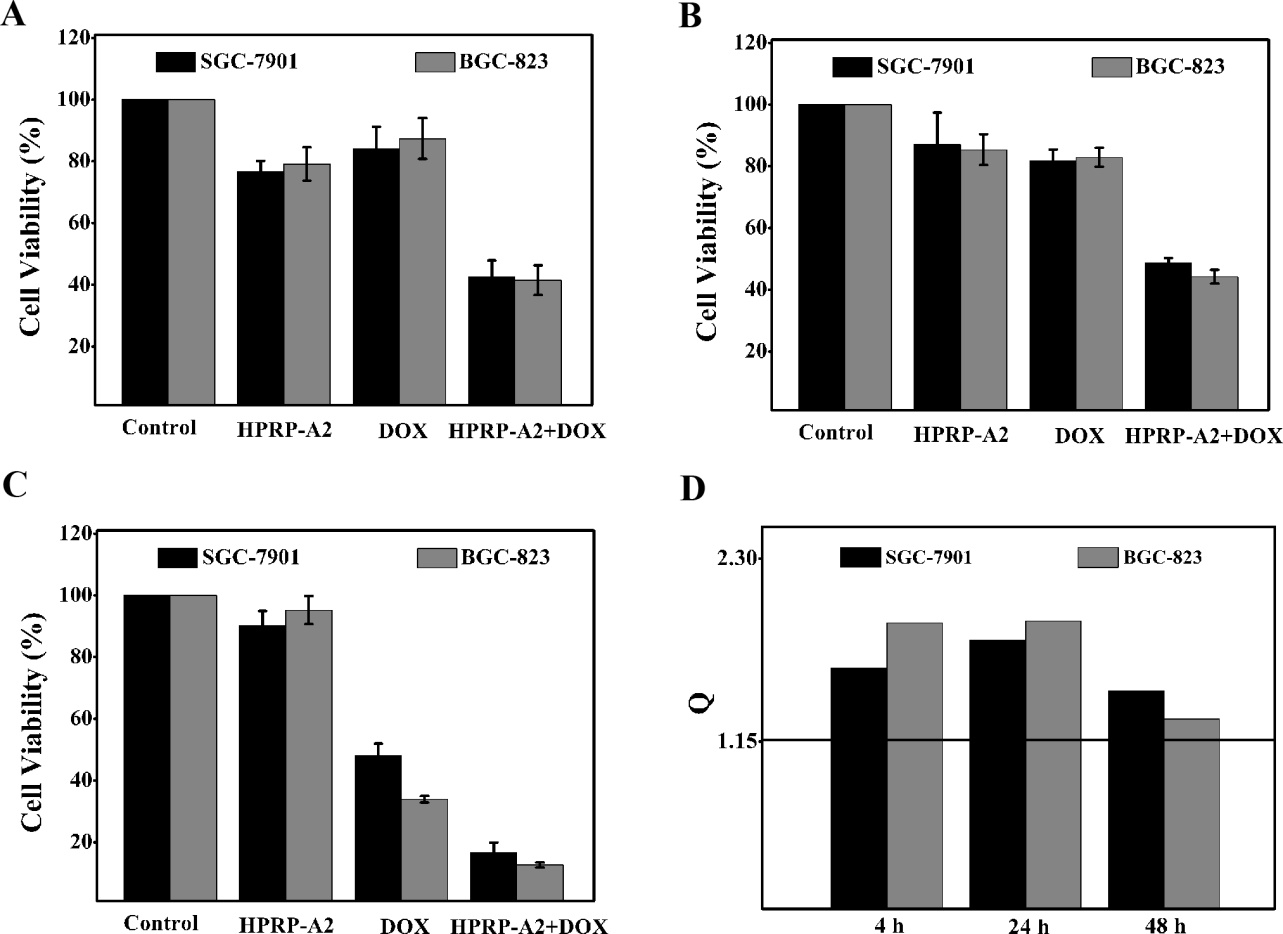
Cell viability and combination index of BGC-823 and SGC-7901 treated with a drug combination. Panel (A, B and C) represents growth inhibition in BGC-823 and SGC-7901 cells with a combination of HPRP-A2 (6 μM) and DOX (1.6 μg/ml) after incubation for 4, 24 and 48 hours, respectively. Results are expressed as the percentage of the control ± SD of three independent experiments. Panel D shows combination index (Q) of the combination treatment of HPRP-A2 and DOX, where Q<0.85, Q>1.15 and 0.85<Q<1.15 indicate antagonism, synergy, and additive effect, respectively. Statistical analysis compared the combination treatment group with the DOX groups (*P < 0.005; **P <0.001).

BGC-823 and SGC-7901 cells were selected to study the synergistic anticancer effect of HPRP-A2 and chemotherapeutic drug DOX. Cells were treated with HPRP-A2 (6 μM) and/or Dox (1.6 μg/ml) for 4, 24 and 48 h. MTT assays were used to evaluate the combinational anticancer effects on cells. The drug concentrations selected in this study were based on the IC_50_ values of each drug alone. There was no obvious cytotoxicity or growth reduction when each drug was used alone. In contrast, when used in the peptide/drug combinations (HPRP-A2/DOX) at the same doses of being used alone, the combination exhibited significant cytotoxicity (Fig 7). It is also clear that anticancer activity of HPRP-A2 was not much affected by the incubation time; in contrast, with the increase of incubation time to 48 h, DOX shows much greater anticancer activity than that of 4 h, indicating the dramatically different mechanisms of action between ACP and chemotherapeutic drug. According to the Jin’s formula,all Q (combination index) values were greater than 1.15, which indicates that there were significant synergistic effects between the α-helical peptide HPRP-A2 and the conventional anticancer drug DOX in BGC-823 and SGC-7901 cells.

## Discussion

Anticancer peptides (ACPs) recently have received great attentions as promising chemotherapeutic agents that avoid the drawbacks of current drugs. Many studies have verified that some synthetic and natural cationic peptides possess a rapid and broad spectrum of anticancer activity towards tumor cells rather than normal cells such as human red blood cells [4, 15]. Moreover, ACPs were also verified to have ability to overcome the multidrug-resistance mechanism, and synergistic effects in combination treatment [11].

HPRP-A2 possesses a rapid and broad spectrum of anticancer activity, however, some differences also occur in the sensitivity of the HPRP-A2 to different cell lines. Based on the different IC_50_ values for HPRP-A2 to BGC-823 (8.65 ±0.38 μM), SGC-7901 (10.42 ±0.30 μM), PC3 (21.38±0.56 μM), and B16 (19.16±0.38 μM), we chose BGC-823 and SGC-7901 cells as research targets. Besides, the anticancer activities of HPRP-A2 to other cancer cell lines such as HeLa and HepG2 have been published in our previous paper [12]. Both of BGC-823 and SGC-7901 cells belong to gastric cell lines, thus, we selected BGC-823 as an example to investigate the anticancer mechanism of HPRP-A2 *in vitro*.

In this study, we have shown that HPRP-A2 is an amphipathic α-helical peptide with significant anticancer activity to BGC-823 and SGC-7901 cell lines. Our studies have indicated that HPRP-A2 exhibited a cancer-selective toxicity, mainly because that cancer cells are composed of more anionic phospholipids and contain O-glycosylated mucin, which increases the negative charge on the cancer cell surface [16, 17]. Moreover, more microvilli on cancer cells can increase the concentration of binding peptide by expanding the membrane surface and thereby show stronger cytotoxicity against cancer cell membranes [11, 18].

ACPs are capable of disrupting cell membrane, which may cause cell membrane permeability changes. In this study, the change of BGC-823 cell membrane was observed by detecting PI-permeabilization and LDH release. The gradual increases of PI permeabilization and LDH release are in concentration-dependent manners after the treatment with HPRP-A2. Moreover,HPRP-A2-induced cell death is associated with the generation of reactive oxygen species, the depolarization of MMP, the activation of the caspase activities and the block in G1 phase of cell cycle.

In addition to the cytotoxicity of HPRP-A2, we proved that it could increase the efficacy of DOX against BGC-823 and SGC-7901 cells, which is consistent with our previous study. In our previous study, we have explored combinational anticancer therapy using α-helical peptides HPRP-A1 and HPRP-A2 with the chemical drugs DOX and EPI in HeLa and HepG2 cell lines [12]. The synergy shown permits the uses of relatively low concentrations of peptides and drugs to achieve significant anticancer effects *in vitro* and *in vivo*. This dose reduction minimizes drug side-effects on normal cells and enables an effective apoptosis-mediated anticancer effect. Our present study has implications in that HPRP-A2 may become a promising anticancer therapeutic agent with high anticancer selectivity and strong synergistic effect in combination therapy. Our studies mainly illustrate the mechanism of HPRP-A2-induced cell death and may be helpful in design of chemotherapeutics against gastric cell lines.

## Conclusions

HPRP-A2 shows strong anticancer activity to BGC-823 and SGC-7901 cell lines and low toxicity against human red blood cells. HPRP-A2 induced cancer cell death through both direct membrane-destructive effect and intracellular mechanisms, including a dramatic increase in caspase-3, -8 and -9 activation, a reduction of mitochondrial membrane potential (MMP), and the generation of ROS and cell cycle arrest in G1. Besides, HPRP-A2 synergized strongly with DOX to enhance the efficacy of killing gastric tumor cells *in vitro*. Our results underscore the broad anticancer potential of HPRP-A2 and elucidate its mechanism of action. We believe that endowing ACPs with more effective and tumor-targeting properties will open up new ways to combat cancer successfully.
